# Five nanometer size highly positive silver nanoparticles are bactericidal targeting cell wall and adherent fimbriae expression

**DOI:** 10.1038/s41598-022-10778-9

**Published:** 2022-04-25

**Authors:** Lok R. Pokhrel, Zachary L. Jacobs, Dmitriy Dikin, Shaw M. Akula

**Affiliations:** 1 Department of Public Health, The Brody School of Medicine, East Carolina University, Greenville, NC USA; 2 School of Law, University of California, Berkeley, Berkeley, CA USA; 3 Department of Mechanical Engineering, College of Engineering, Temple University, Philadelphia, PA USA; 4 Department of Microbiology and Immunology, The Brody School of Medicine, East Carolina University, Greenville, NC USA

**Keywords:** Infectious diseases, Toxicology, Drug discovery, Microbiology, Health care, Medical research, Nanoscience and technology

## Abstract

To tackle growing antibiotic resistance (AR) and hospital-acquired infections (HAIs), novel antimicrobials are warranted that are effective against HAIs and safer for human use. We hypothesize that small 5 nm size positively charged nanoparticles could specifically target bacterial cell wall and adherent fimbriae expression, serving as the next generation antibacterial agent. Herein we show highly positively charged, 5 nm amino-functionalized silver nanoparticles (NH_2_–AgNPs) were *bactericidal*; highly negatively charged, 45 nm citrate-functionalized AgNPs (Citrate–AgNPs) were *nontoxic*; and Ag^+^ ions were *bacteriostatic* forming honeycomb-like potentially resistant phenotype, at 10 µg Ag/mL in *E. coli*. Further, adherent fimbriae were expressed with Citrate–AgNPs (0.5–10 µg/mL), whereas NH_2_–AgNPs (0.5–10 µg/mL) or Ag^+^ ions (only at 10 µg/mL) inhibited fimbriae expression. Our results also showed no lipid peroxidation in human lung epithelial and dermal fibroblast cells upon NH_2_–AgNPs treatments, suggesting NH_2_–AgNPs as a biocompatible antibacterial candidate. Potent bactericidal effects demonstrated by biocompatible NH_2_–AgNPs and the lack of toxicity of Citrate–AgNPs lend credence to the hypothesis that small size, positively charged AgNPs may serve as a next-generation antibacterial agent, potentially addressing the rising HAIs and patient health and safety.

## Introduction

Hospital-acquired infections (HAIs), synonymously called nosocomial infections, are detrimental to patient safety and recovery. HAIs are a threat in all hospitals, but the intensive care unit (ICU) documents the highest HAI rates^1^. Moreover, HAIs are most common with the central line bloodstream and ventilator usage costing an extra 9.5 and 9.1 days of hospital stay, respectively^2^. Annually, over 12 million deaths occur due to HAIs globally, of which 95% occur in low- and middle-income countries (LMICs)^3^. In the United States alone, over 2 million infections are caused by HAIs, particularly associated with antibiotic resistance (AR) and/or multidrug-resistant organisms (MDROs), of which about 23,000 fatalities occur, annually^2^.

Gram-negative bacteria are considered more resistant to current antibiotics compared to gram-positive bacteria, mainly due to the presence of cell wall that limits the uptake of antibiotics in the former and is lacking in the latter^4^. *Escherichia coli* is among the gram-negative bacteria commonly causing HAIs^5^, including food-borne outbreaks^6^, and ultimately deaths^5^. In virulent strains, such as *E. coli* O1:K1:H7 (CN1018), expression of adherent fimbriae has been associated with increased bacterial survival and inflammatogenic response in the urinary tract infection mouse model^7^. Moreover, bacterial isolates lacking expression of fimbriae have demonstrated reduced growth, adhesion, biofilm formation and virulence, thereby resulting in low infections and improved survival in animal models and humans when infected with the virulent strains^8^. Hence, a need for better antimicrobial agents that can specifically target the cell wall and/or adherent fimbriae expression cannot be overstated, and engineered nanomaterials (ENMs) may have the potential to serve as the next generation antimicrobial agent^9^.

ENMs, particularly silver-based nanoparticles (AgNPs), are being widely used in various commercial applications (e.g., fabric, mask, medical device, paint, cement, water disinfection, etc.), but the potential factors and mechanisms underpinning their antimicrobial activities have remained unclear^10–13^ as results are largely compounded by the heterogeneity of the ENMs used, endpoints measured, and differential sensitivity of the bioassays used. As the studies on the actual mode of ENM toxicity is ongoing, recent literature suggest that the capping ligand, core particle size, and surface charge likely play a role^14–18^. In the recent rule published by the USEPA under TSCA (Toxic Substances Control Act), the roles of inherent particle attributes, including the particle size, surface charge (measured as zeta potential), aggregation state, or surface reactivity on ENM toxicity, were emphasized as factors commonly linked to nanotoxicity^19^. Necessary for future endeavors is an understanding of desired ‘nano’ properties, so certain characteristics may be wishfully tailored to render ENM safer (e.g., for environmental remediation) or more toxic (e.g., for neutralizing AR/MDROs).

Molecular or atomic scale interactions occurring at the nanoscale was previously difficult to ascertain optically owing to the Abbe diffraction^20^. Nonetheless, routine use of electron microscopy in tandem with surface analytical spectroscopic techniques have now enabled researchers to track individual NPs on the cell surface and its interior (cytoplasm and nucleus). Recently, Werner and colleagues^20^ asserted that “it would be advantageous to require the nanomaterial to be as minimally specific as necessary in order to act as a delivery vector, a nanosensor, or an imaging agent.” This is crucial because a given NP has several physico-chemical properties, among which only a few characteristics are routinely measured, including particle size, zeta potential, surface linker, aggregation, etc. Adhering to the ‘Occam’s razor’, also called ‘philosophy of parsimony’^21^, a minimal set of defined physicochemical properties of ENMs that could be routinely measured may enable designing ENMs for desired applications such as treating the HAIs. Previous studies point to the fact that AgNPs may act as an antibacterial agent, but the mode of action (MoA) has largely remained nebulous^10,11^. Herein, employing two different sized (5 nm versus 45 nm) AgNPs with high contrasting surface charges (high positive versus high negative), including dissolved ionic Ag^+^ as positive control, this study aimed at: (1) elucidating potential factors and mechanisms influencing nano-bio interactions and toxicity in *E. coli*, a common gram-negative bacillus with high AR/MDR potential; (2) using this information, determining the most potent antibacterial AgNPs; and (3) documenting potential phenotypic plasticity (i.e., shape-shifting) in *E. coli* in response to different silver treatments. The results of this study are novel, demonstrating higher potency of small size and highly positive charged NH_2_–AgNP, via cell wall damage and adherent fimbriae inhibition compared to the larger size and negatively charged Citrate–AgNPs, and that NH_2_–AgNPs are biocompatible or safer to human cells, highlighting its potential application as a next-generation bactericidal agent capable of addressing the rising HAIs and patient health and safety.

## Experimental

### Synthesis and purification of silver nanoparticles

Two different types of silver nanoparticles (AgNPs) were synthesized and used in this study: (1) amino (NH_2_)-functionalized, positively charged AgNPs (NH_2_–AgNPs; patent #PCT/US2021/014,343)^21^ of average TEM diameter of 5.8 nm, and (2) citrate-functionalized, negatively charged AgNPs (Citrate–AgNPs) of average TEM diameter of 44.8 nm^22,23^. All chemicals used in this study were of highest purity and procured from Fisher Scientific. The NPs were synthesized in the laboratory using the protocols that follow: For NH_2_–AgNPs synthesis, 1 mL ethyleneimine (~ 1 g) and 1.19 g HEPES were dissolved in Milli-Q water and brought the volume to 500 mL. In a separate flask, 170 mg of silver nitrate (AgNO_3_) and 1.19 g HEPES were added together, and brought the volume to 500 mL using Milli-Q water. The contents of both flasks were then added to a liter flask and mixed thoroughly for 30 min. Using two UV lights (λ_254 nm_), the solution was irradiated for 6 h under constant stirring (30 rpm). The solution was then heated to 90 °C for 45 min under constant stirring (30 rpm), then removed from the heat source and added 7.5 mg of borohydride. The suspension was then left at room temperature to cool overnight. Likewise, Citrate–AgNPs were synthesized by adding 1 mM AgNO_3_ to 10 mM sodium citrate in a volume ration of 2:1. The solution was then heated for 4 h at 50 °C in a water bath and was left to cool at room temperature overnight. Upon synthesis, both the NPs were purified using a tangential flow filtration (TFF) system equipped with 3.5 kD hollow fiber polysulfone membranes as we previously described (details in Supplementary Information Table S1)^12,24^.

### Characterization of nanoparticles

Transmission electron microscopy (TEM, Philips EM 420, 120 kV, brightfield mode) was used to determine particle size, particle size distribution, and shape of the AgNPs tested. High-resolution Field Emission-Scanning Electron Microscopy (FEI Quanta 450 FEG) was employed for imaging *E. coli* cell surface morphology and visualizing potential NP interactions with the *E. coli* surfaces. Samples were drop-casted on to the double-sided adhesive carbon tape attached to the aluminum stub for standard reflective-mode microscopy and on to copper grid with formvar coating for transmissive-mode SEM (STEM), and allowed to dry at room temperature while any excess suspension was absorbed along the edge using a blotting paper. A low vacuum imaging regime (40–80 Pa) of water vapor was applied during the reflective-mode imaging in order to neutralize potential sample charging. *E. coli* surfaces were then scanned by electron beam using a low accelerating voltage of 3–5 kV for reflective imaging, and 30 kV for transmissive imaging, with a working distance of around 10 mm. Upon EM analysis, energy dispersive spectroscopy (EDS) analysis was performed on the AgNPs and *E. coli* surfaces for assessing potential nano-bio interactions using AZtec Energy Advanced with X-Max SDD 50 sq mm, 127 eV resolution detector (Oxford Instruments). EDS information was collected in three modes: Point mode, ID mode and Mapping mode.

UV–Vis Spectroscopy (Hach DR6000) was used to measure the localized surface plasmon resonance, λ_max_ (maximum wavelength at which the plasmonic peak was observed), of the AgNPs. Hydrodynamic diameters (HDDs) of the AgNPs were determined using the dynamic light scattering (DLS; Zetasizer Nano ZS90, Malvern Panalytical) and Smoluchowski equation was used to estimate zeta (ζ) potential of the AgNPs based on electrophoretic mobility of the NPs using the ζ potential software.

### Toxicity bioassay and treatment conditions

*Escherichia coli* dh5a was used as a model test bioassay. *E. coli* is a gram-negative bacillus that reproduces by binary fission^25^. Luria–Bertani (LB) medium was used as a culture and growth medium for *E. coli*. The LB broth constituted: 10 g peptone, 5 g yeast extract, and 5 g NaCl. 6.25 g LB powder was mixed with 250 mL of ultrapure water in a beaker. The solution was stirred to mix uniformly and autoclaved for 15 min at 121 °C before use for the bioassay. Four mL of the prepared LB broth was pipetted into a five mL centrifuge tube. Then each tube received either 0.5 mL bacteria + 0.5 mL water, which served as a negative control, or 0.5 mL bacteria + 0.5 mL AgNPs (of each type) as the treatment. Two concentrations, 0.5 µg/mL and 10 µg/mL, covering low and high exposure conditions, respectively, were tested for both types of AgNPs with differing particle sizes, surface charges and coating materials. In addition, we also tested potential effects of ionic silver (Ag^+^ in Milli-Q water) at the same concentrations used for AgNPs (0.5 µg/mL and 10 µg/mL) for comparison. Initial *E. coli* cell density was maintained at 10^5^–10^6^ cells/mL. Bacteria were then incubated at 37 °C and population growth was measured as optical density (OD) using UV–Vis spectrophotometer at 600 nm as a function of time (0, 4, 24, 48, and 72 h); experiments were halted at 72 h. Before each measurement, samples were gently vortexed for 3 s to improve measurement accuracy. Each experiment was repeated twice, and data reported as aggregates. Data were corrected for background absorbance.

Aliquots of *E. coli* test samples were further used for STEM-EDS analyses to assess AgNP-cell interaction, AgNP localization on the cell surface, cell surface elemental mapping, potential cell damage, and/or morphological alterations in *E. coli* upon AgNPs exposure.

### Biocompatibility assay

Reactive oxygen species (ROS) may be generated upon NP exposure. Lipid peroxidation occurs when excess ROS affects cell membranes and can also lead to oxidation and denaturation of proteins and DNA damage, further inducing inflammatory immune responses and cell death^26^. We tested potential lipid peroxidation (Malondialdehyde [MDA] assay) in human lung epithelial (H-6053; Cell Biologics) and dermal fibroblast (106-05A-1526; Millipore Sigma) cells upon NH_2_–AgNPs treatments (0.05–10 µg/mL) to assess its biocompatibility with human cells in vitro as per standard manufacturer’s protocol (Abcam; ab118970)^27^. The sample absorbance was read at 532 nm using a microplate reader. Each reaction was performed in triplicate. Sterile water (dilution buffer) was used as a negative control and hydrogen peroxide (200 µM) was used as a positive control.

### Statistical analysis

*Escherichia coli* morphological dimensions, length and diameter quantified from SEM images using ImageJ were tested for equality of variances using Levene’s test before conducting independent samples t-test (2-tailed), which tested for significant differences between the treatment sample means and the controls at *p* ≤ 0.05 level. Further, using General linear model we tested if cell diameter was a predictor of cell length under different treatment conditions for *E. coli*. We found that cell diameter could adequately predict cell length in *E. coli*; we then used centroid plot to visualize the distinct *E. coli* clusters based on the overall cell dimensions (length vs. diameter). Statistical analyses were conducted using IBM SPSS (version 16; Chicago, IL, USA).

### Quality assurance

All containers used in this study were soaked in 5% HNO_3_ overnight, cleaned several times using Milli-Q water (18.2 MΩ-cm, TOC < 10 ppb), and air dried before use. Electron microscopes are routinely calibrated as part of good laboratory practices.

## Results and discussion

**Figure 1:**
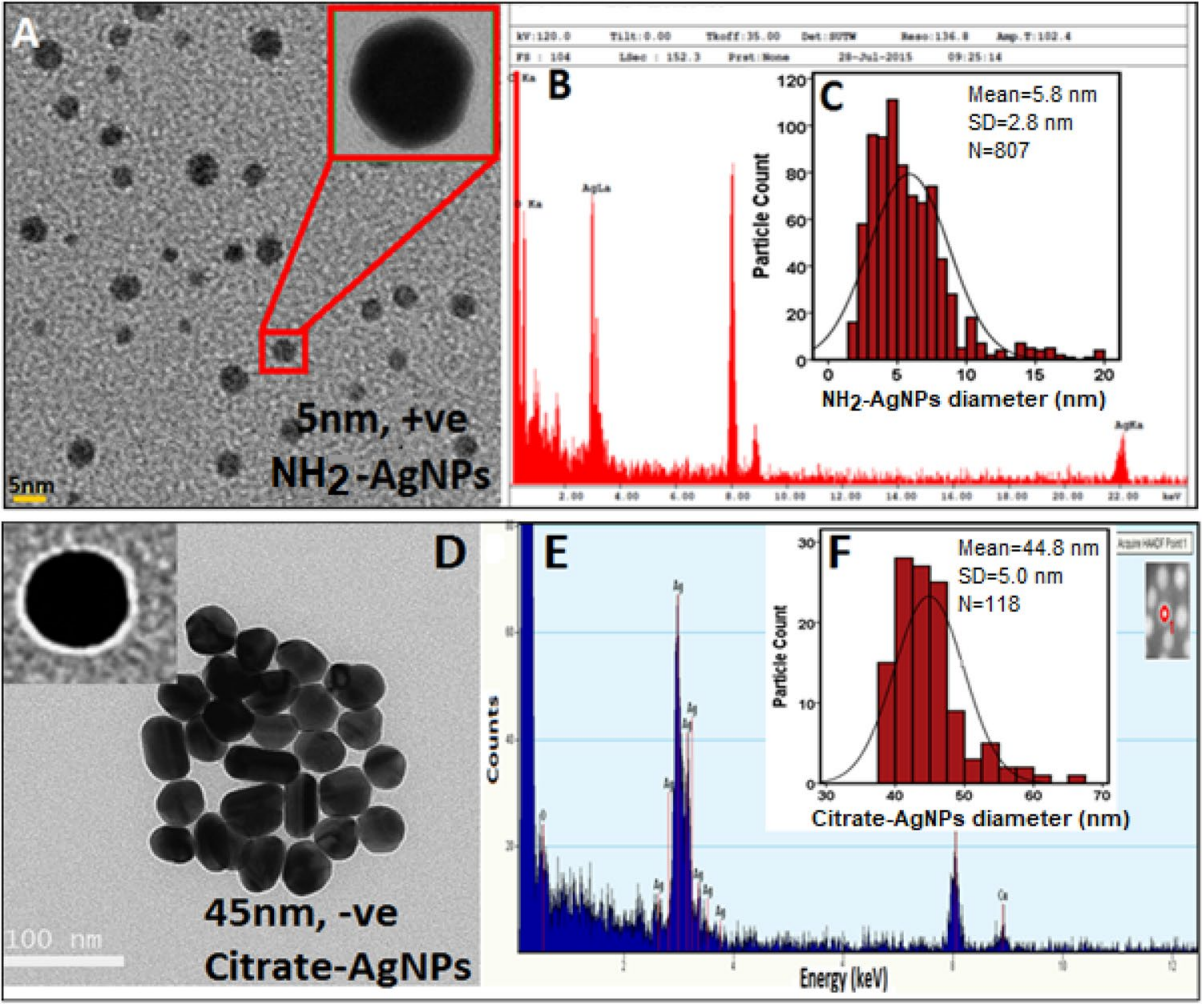
Transmission electron microscopy (TEM) image (**A**), energy dispersive spectroscopy (EDS) (**B**), and particle size distribution (PSD) (**C**) analyses of 5 nm diameter, positively charged amino-functionalized silver nanoparticles (NH_2_–AgNPs). TEM image (**D**), EDS (**E**), and PSD (**F**) analyses of 45 nm diameter, negatively charged Citrate-functionalized silver nanoparticles (Citrate–AgNPs).

Purified AgNPs were characterized using the TEM and images were analyzed using an ImageJ. Average TEM diameter of the NH_2_–AgNPs was 5.8 ± 2.8 nm, and were spherical in shape, crystalline, and had a high positive average zeta potential of + 41.6 mV due to the cationic amino groups on the NP surface (Fig. 1; Table 1). The uniform NH_2_– coating thickness on the surface of AgNPs was measured in the range 0.5–1.5 nm, thereby enabling AgNPs’ stability via electrosteric repulsion. Average TEM diameter of Citrate–AgNPs was 44.8 ± 5.0 nm, and were pseudospherical in shape, crystalline, and had a high negative average zeta potential of −30 mV due to the anionic carboxyl groups on the NP surface (Fig. 1; Table 1). The uniform citrate-coating thickness on the surface of AgNPs was 1.8 nm, which enabled NPs stability via electrostatic stabilization. Further, TEM analysis of AgNPs morphology suggests that both the NH_2_– and citrate-ligands could effectively confer stability to AgNPs by preventing aggregation, and thus the nano-suspensions were observed to be highly stable in aqueous suspension over several years (> 3 yrs.). Dynamic light scattering (DLS) analysis of average hydrodynamic diameter (HDD) for NH_2_–AgNPs was 4.3 nm and 11 nm for Citrate–AgNPs. As previously documented, the observed disparity in the diameter of Citrate–AgNPs by DLS and TEM analyses might be ascribed to the underlying differences in the measuring principles used by TEM and DLS methods^12,23^. UV–Vis spectrophotometry analysis showed localized surface plasmon resonance (λ_max_) at 416.5 nm for NH_2_–AgNPs, and 425 nm for Citrate–AgNPs, and that dilution (Supplementary Information Fig. S1), incubation time (72 h) and temperature (35 °C) had no effect on the stability of both the AgNPs (Supplementary Information Table S2).

**Table 1:** Impacts of differential surface charge of two AgNPs types on nano-bio interactions and toxicity in *E. coli* dh5a.

Samples	Mean Zeta potential^a^ (mV)		Magnitude of charge difference^b^ |A-B|	Strength of interaction	Expected toxicity	Observed toxicity
	*Day-0 (A)*	*Day-3* (w/ *E. coli*) *(B)*				
*E. coli*	−14.8	−10.0^c^	4.8	na	na	na
Citrate–AgNPs	−30.0	−15.0	15.0	Lower electrostatic attraction	Lower	Lower
NH_2_–AgNPs	+ 41.6	−17.0	58.6	Higher electrostatic attraction	Higher	Higher

^a^Zeta potential measured in test media (LB broth).

^b^Magnitude of charge difference is presented as an absolute value.

^c^Minor change in zeta potential of control samples (*E. coli* + LB broth) over 72 h period.

‘Cit–AgNPs’ denotes citrate-functionalized AgNPs; ‘NH_2_–AgNPs’ denotes amino-functionalized AgNPs. ‘na’ denotes not applicable.

**Figure 2:**
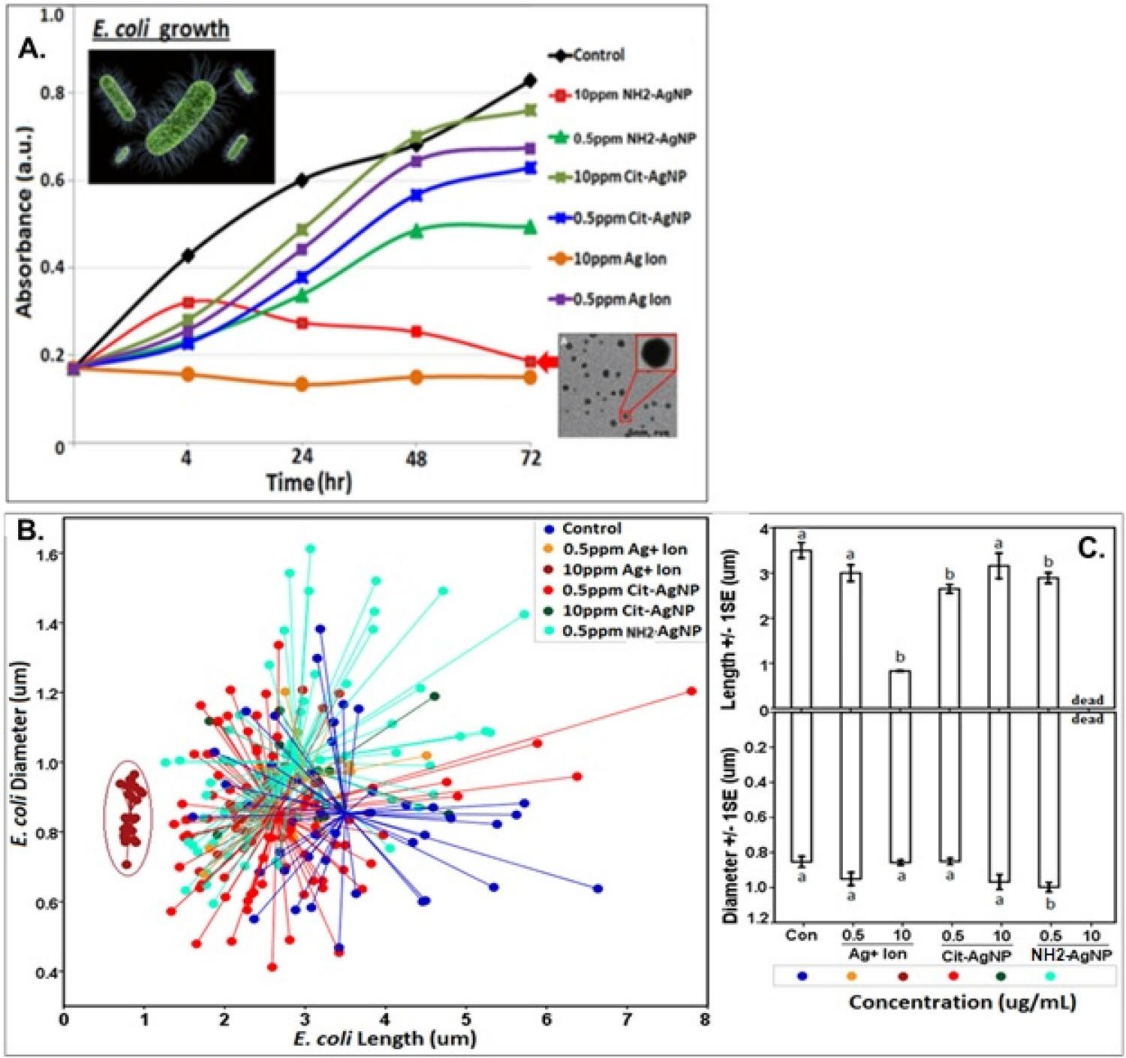
Variation in *E. coli* dh5a growth under different treatment conditions and incubated at 37 °C over a period of 72 h. Treatment data were corrected for background absorbance. Ag^+^ ions showed *bacteriostatic effect* at 10 µg Ag/mL; NH_2_–AgNPs showed *bactericidal effect* at 10 µg Ag/mL, while other treatments led to increased growth of bacterial population as a function of time (**A**). *E. coli* diameter (Y-axis) plotted against its length (X-axis) depicting morphological alterations in cell size under different treatment conditions (**B**); each data point is connected to the centroid showing discrete morphological clusters under different treatments (**B**). Significant variation in bacterial cell length and diameter were tested using two-tailed t-test at *p* ≤ 0.05 (**C**). Same letter above the bar indicates no significant difference between the sample means. Corresponding SEM images of the *E. coli* cells upon different silver treatments are presented in Fig. 3.

Our results showed that *E. coli* population increased over the 72 h period for the negative controls (Fig. 2). Citrate–AgNPs (0.5 or 10 µg/mL), or Ag^+^ ions particularly at 0.5 µg/mL, also showed positive cell growth (OD_600_ > 0.6 a.u.) over 72 h. However, exposure to 10 µg/mL NH_2_–AgNPs showed, after initial acclimation, a steady decline in *E. coli* growth with population completely crashing by 72 h, confirming a *bactericidal* effect of NH_2_–AgNPs at 10 µg/mL (Fig. 2A; red scatter line). Exposure to 10 µg/mL Ag^+^ ion demonstrated strong *bacteriostatic* effect as the growth curve showed a flat line (Fig. 2A; orange scatter line). Further, at a low concentration of 0.5 µg/mL NH_2_–AgNPs inhibited bacteria growth more effectively than the same concentration of Citrate–AgNPs or Ag^+^ ions (Fig. 2). Taken together, these results indicate that NH_2_–AgNPs possess strong *bactericidal* activity, while Ag^+^ ions have strong *bacteriostatic* effect, at 10 µg Ag/mL level.

**Figure 3:**
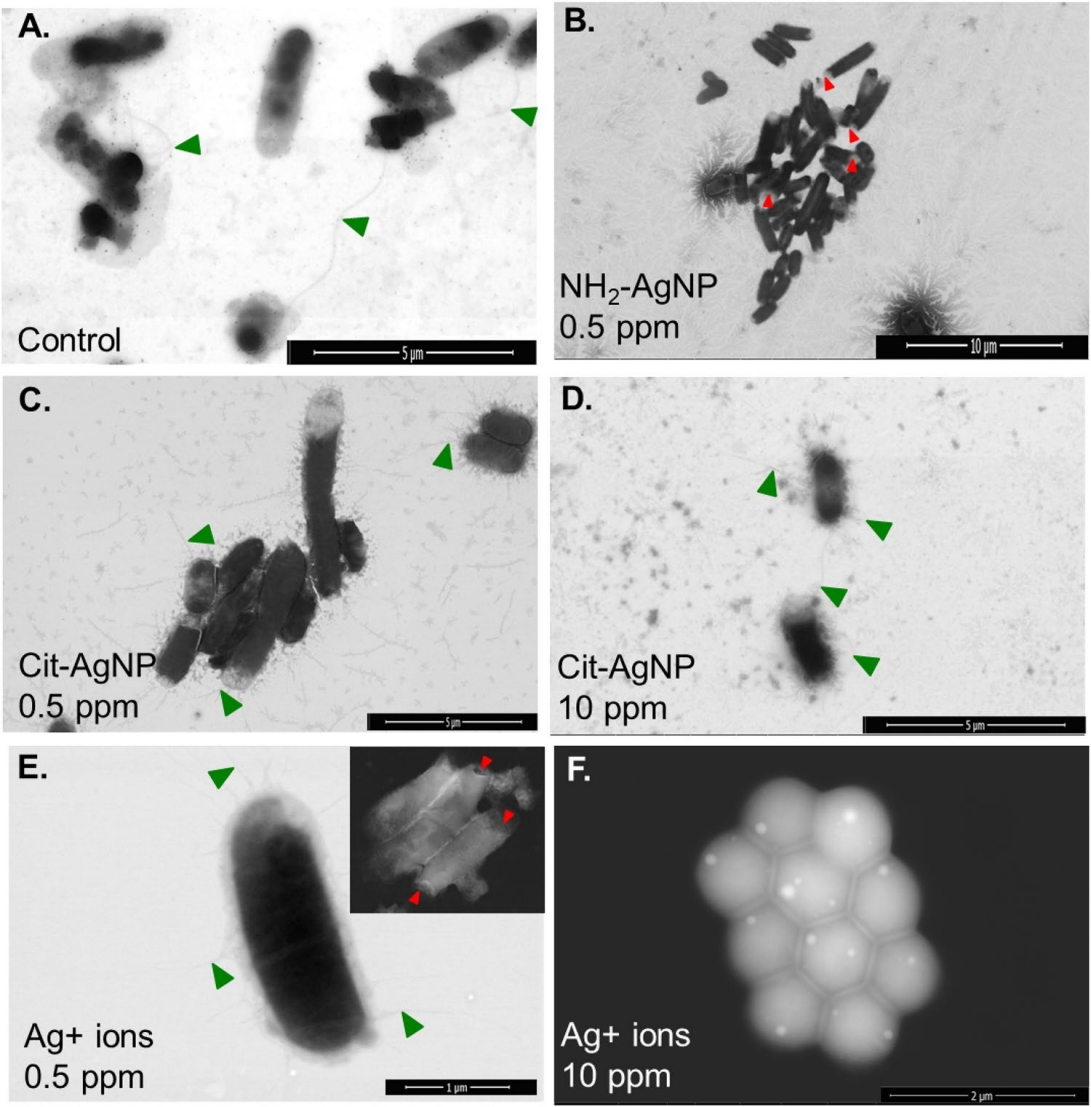
Representative Field Emission-Scanning Electron Microscope (FE-SEM) images of *E. coli* dh5a imaged after 72 h of exposure to different Ag treatments: (**A**) Control; (**B**) NH_2_–AgNPs, 0.5 µg/mL; (**C**) Cit–AgNPs, 0.5 µg/mL; (**D**) Cit–AgNPs, 10 µg/mL; and (**E**) Ag^+^ ions, 0.5 µg/mL; and (**F**) Ag^+^ ions, 10 µg/mL. Note, NH_2_–AgNPs at 10 µg/mL treated cells are not shown as no bacteria could be located under the FE-SEM after 72 h because the cells were likely disintegrated and dissolved upon exposure. Green triangle denotes adherent fimbriae being expressed, and red triangle denotes cell wall damage. ‘ppm’ denotes parts per million, equivalent to µg/mL.

**Figure 4:**
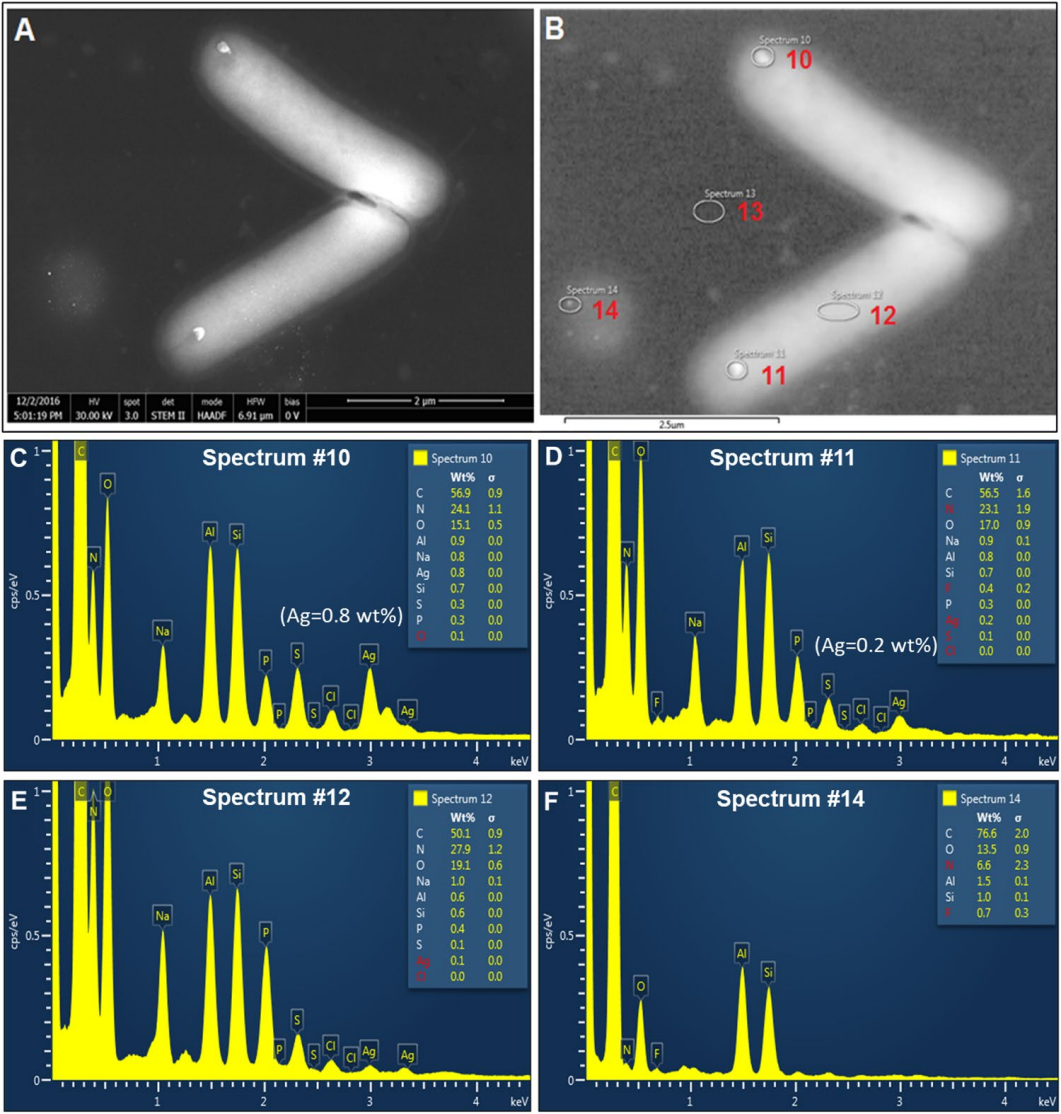
Field Emission-Scanning Electron Microscopy (FE-SEM) analysis of *E. coli* morphology upon exposure to 10 µg/mL NH_2_–AgNPs for 10 min (**A, B**) revealed a larger nanoparticulate of Ag (size range 155–157 nm in diameter) at the end of each cell surface. EDS spectra (spectra #10–14) of Ag across the selected areas on *E. coli* surfaces were recorded (**C-F**), which confirmed that the nanoparticulates observed at the end of cells were composed of elemental Ag (**C, D**).

Potential interaction with the bacterial cell wall upon physical contact with NPs is a plausible explanation for antibacterial effects of AgNPs^12^. Small size (5 nm diameter) AgNPs could competitively interact with bacterial cell wall due to electrostatic attraction (Fig. 4A). Further, high positively charged cationic AgNPs (with amino [-NH_2_^+^] as surface ligand) demonstrated higher affinity to bacterial cell surface binding compared to the negatively charged AgNPs (with citrate [-COO^-^] moiety) (Figs. 3 and 4).

**Figure 5:**
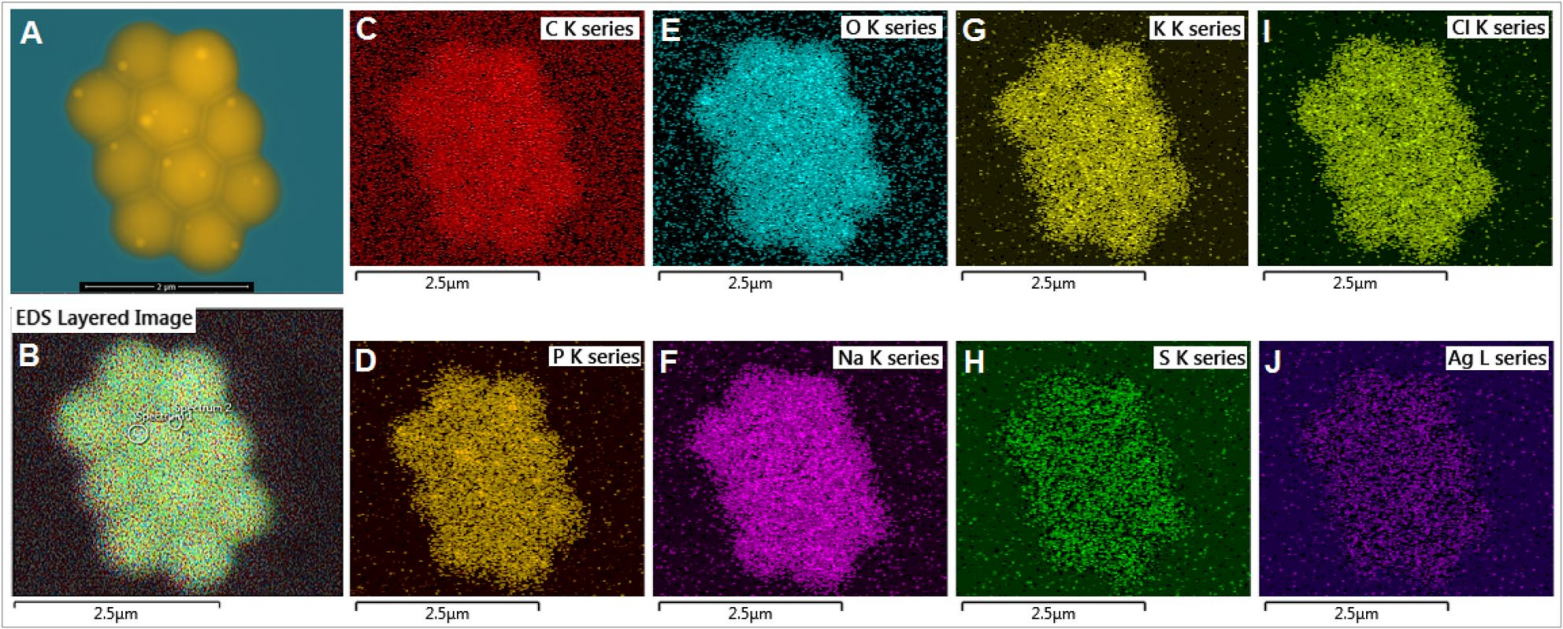
Energy dispersive spectroscopy (EDS) elemental mapping of silver on *E. coli* dh5a surfaces upon exposure to 10 µg/mL Ag^+^ ions for 72 h. Scale bar denotes 2.5 µm for all images, except for A (scale bar = 2 µm).

We further assessed if *E. coli* cell diameter is a predictor of cell length using the General Linear Model. Results showed that cell diameter is a strong predictor of cell length (Supplementary Information Table S3). Plotting cell diameter versus cell length (Fig. 2B, C) revealed distinct cell clusters reflecting differing cell morphologies. Cells treated with 10 µg/mL Ag^+^ ions demonstrated change in cell morphology from individual rod-shaped to hexagonal honeycomb-like clusters of cells stacking together (Figs. 3F, 5). Such hexagonally stacked honeycomb phenotype is known to minimize surface area to volume ration, thereby significantly reducing available exposure surfaces per bacterial cell to the stressor, and in this case, Ag^+^ ions at 10 µg/mL^28^. Moreover, it has been suggested that hexagonal honeycomb structure, as observed in beehives, is likely the most economically feasible shape or form in terms of material and energy expended to form or build the honeycomb^28^. To our knowledge, this is the first study to report a honeycomb-like, potentially resistant cell phenotype in gram-negative *E. coli* upon Ag^+^ ions exposure. At 10 µg/mL NH_2_–AgNPs treatment, *E. coli* survival severely diminished before the population completely crashed by 72 h as verified by FE-SEM analysis (i.e., no cell could be located in the samples under the FE-SEM). This result confirms potent *bactericidal* effects of NH_2_–AgNPs at 10 µg Ag/mL, thereby averting the evolution of potentially resistant phenotype as observed for Ag^+^ ions.

It was recently documented that bacteria could form lenticular-shaped chains upon metal stress. Chakravarty & Banerjee^29^ studied morphological changes in *Acidiphilium symbioticum* H8 upon exposure to Cu (as 500 mM CuSO_4_) and Cd (as 12.5 M CdSO_4_) and found that loosely packed coccobacillus-type cells (short rod-shaped) transformed into round shape and were highly packed together upon exposure to the metals. Likewise, Khusro et al.^30^ noted similar stress response induced in *Bacillus subtilis* KPA upon exposure to mercuric chloride, wherein treated cells presented round phenotype from the native rod shape.

Because bacteria exposed to 10 µg/mL NH_2_–AgNPs for 72 h did not survive and thus the cells could not be located under the SEM, we then briefly exposed the bacteria for 10 min to the same concentration (10 µg/mL) of NH_2_–AgNPs and mapped AgNPs-cell interaction, potential cell wall damage, and elemental Ag validation across the selected areas as shown in Fig. 4. SEM analysis of the bacterial cells did not reveal morphological damage, but a larger particulate of Ag (size range 155–157 nm in diameter) was clearly found to be located at the end of each cell (see Energy Dispersive Spectroscopy [EDS] spectra #10 and #11 in Fig. 4C,D). It was previously shown that smaller size and positively charged AgNPs are relatively more toxic that the larger size and negatively charged AgNPs^12^, but the NH_2_–AgNPs’ localization at the end of *E. coli* cells has been for the first time clearly imaged in this study at high resolution using the STEM mode. It can thus be inferred that 5 nm diameter, highly positively charged NH_2_–AgNPs precipitated upon electrostatic interactions with the bacterial cell surfaces and/or biomolecules within the cell exterior, thereby forming larger particulates of Ag ranging in diameter 155–157 nm (Fig. 4A,B). Furthermore, high positively charged NH_2_–AgNPs attachment to the bacterial cell surface may lead to “hyperpolarization” of the cell wall, more specifically, peptidoglycan that makes up the cell wall, as described recently for gold nanoparticle interactions with *E. coli* K12^31^, subsequently leading to adherent fimbriae inhibition and cell wall damage as observed in our study (Figs. 3 and 4, Table 2).

**Table 2:** Toxicity of different AgNPs types on cell wall, adherent fimbriae expression, and shape change in *E. coli* dh5a. Significant effects are shown in bold.

Test chemical	Concentration	Cell damage	Fimbriae	Shape
Control	0	No	Present	Rod
Cit**–**AgNPs	0.5 µg/mL	No	Present	Rod
	10 µg/mL	No	Present	Rod
NH_2_**–**AgNPs	0.5 µg/mL	**Yes**	**Absent**	Rod
	10 µg/mL	**All dead**	**Absent**	**All dead**
Ag^+^ ions	0.5 µg/mL	**Yes**	Present	Rod
	10 µg/mL	No	**Absent**	**Hexagonal honeycomb**

EDS surface elemental mapping of *E. coli* exposed to 10 µg/mL Ag^+^ ions for 72 h showed a greater amount of silver on the cell surface and were found associated with common biological elements such as C, O, Na, K, Cl (Fig. 5). In comparison, *E. coli* exposed to 10 µg/mL Citrate–AgNPs for 72 h confirmed the above toxicity results showing lesser amount of silver on the cell surface and were found associated with such biological elements: C, O, Na, K, and Cl but not with P and S (Supplementary Information Fig. S2).

In *E. coli* dh5a, mannose resistant Proteus-like [MR/P]) fimbriae are expressed^7^. FE-SEM image analyses revealed the presence of filamentous adherent fimbriae in the negative control group, including in both concentrations of Citrate–AgNPs and low concentration (0.5 µg/mL) of Ag^+^ ions (Fig. 3; Table 2). However, adherent fimbriae were completely absent in *E. coli* when treated with NH_2_–AgNPs at both concentrations (0.5 and 10 µg/mL), changing them into non-motile phenotype (Fig. 3B,4A; Table 2). Fimbriae were also absent with Ag^+^ ions treatment, particularly, at 10 µg/mL, when bacteria changed their morphology to hexagonal honeycomb-like phenotype (Fig. 3F; Table 2). Fimbriae expression in pathogenic bacterial strains such as *E. coli* is known to promote adhesion to substrates, biofilm formation, and virulence, causing higher infections and reduced survival in animals and humans^7,8^.

Particle size vis-à-vis material-specific properties could govern nano-bio interactions^12–16,20,32^. Previous studies that modeled artificial phospholipid bilayer interacting with rod-like biomolecules suggest that the energy on the surface may influence surface molecule-cell membrane association, potentially leading to cell entry of biomolecules^32,33^ and toxicity. Energy barrier that exists between NPs and the biologic surfaces ought to be overcome^35^ before the NPs could interact with the receptor surfaces (cell wall or cell membrane). In our study, a remarkable difference in the magnitude of surface charge between the 5 nm NH_2_–AgNPs (ζ =  + 41.6 mV) and 45 nm Citrate–AgNPs (ζ = −30 mV) can be attributed to higher attraction forces between the *E. coli* surface and the NH_2_–AgNPs surface (Table 1), enabling direct physical contact between the two surfaces (as evidenced by EDS spectra showing particulates of silver on the *E. coli* surfaces; see Fig. 4A,B), leading to increased cell death after 4 h followed by complete population collapse by 72 h. On the other hand, a significantly lower charge difference observed for Citrate–AgNPs suggests that the dominant repulsive forces (lower attraction; Table 1) could have played a key role in keeping most of the Citrate–AgNPs away from the biologic surfaces (as confirmed by EDS elemental mapping showing lower amount of silver on *E. coli* surfaces) and this might explain the lowest toxicity observed for Citrate–AgNPs (Figs. 2, 3C,D; Supplementary Information Fig. S2). Evidence from previous studies also corroborate a potential for direct physical interactions between the NPs (e.g., graphene and derivatives) and the biologic receptors^36,37^, suggesting direct physical interaction may serve as a primary mechanism explaining AgNP toxicity.

Shape- and size-shifting in *E. coli* have been known to occur in response to varied environmental stressors^38,39^. It is hypothesized that bacteria can shrink or assume spherical (from originally rod-like) shape to minimize cell surface area when conditions become more stressful^38,39^. Liu et al.^38^ investigated potential effects of ZnONPs (diameter 70 ± 15 nm) in *E. coli* and observed deformed cell membranes that led to cell leakage but did not observe any morphological changes. In another study, AgNPs could potentially penetrate into *E. coli* cells, resulting in membrane degradation^39^. At 24 h post-exposure, a significant growth inhibition was recorded as a function of AgNP concentrations^39^. Formation of irregular-shaped pits in the outer membrane was attributed to inhibition in cell growth and reproduction. Further, increased AgNP concentration resulted in smaller bacterial size^39^. He et al.^40^ treated *E. coli* with MgONPs with an average size of 20 nm. Using SEM, the authors revealed disrupted membranes in *E. coli* exposed to 20 nm MgONPs that led to the rough and leaky *E. coli* cells^40^. Measuring the average roughness of bacteria using atomic force microscopy (AFM) before and after Catechin-CuNPs (5 nm diameter) treatments, Li et al.^41^ found that the untreated *E. coli* cell roughness was 6.32 nm, 4.88 nm (Rq, Ra), whereas for the treated cells the roughness increased significantly to 71.6 nm, 57.3 nm (Rq, Ra); and for *Streptococcus aureus* the roughness increased from 4.51 nm, 3.13 nm (Rq, Ra) to 76.1 nm, 59.9 nm (Rq, Ra). Such evidence strongly supports the premise that NPs (e.g., Catechin-CuNPs) can potentially disrupt the basic cell membrane structure (i.e., form) and consequently the membrane functions^41^.

**Figure 6:**
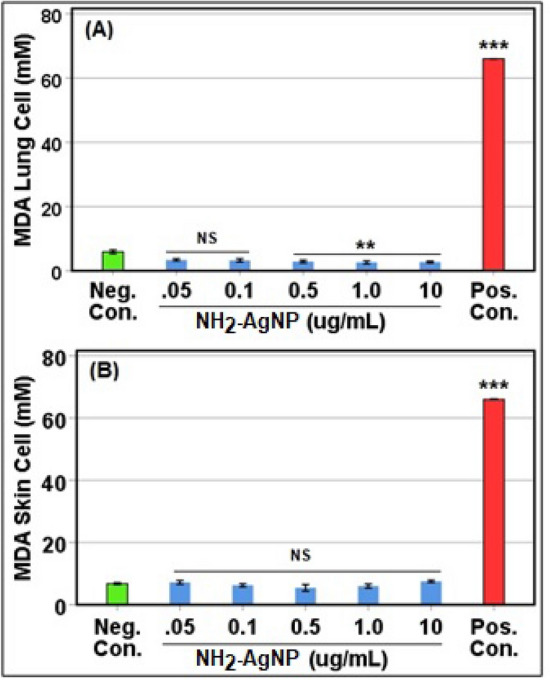
In vitro oxidative stress response of NH_2_AgNPs in primary human lung epithelial (**A**) and dermal fibroblast cells (**B**), showing no Malondialdehyde (MDA) lipid peroxidation in both cell assays. ‘**’ denotes significantly lower compared to negative control (*p* < 0.05); ‘***’ denotes significantly higher compared to negative control (*p* < 0.001); and NS, denotes each treatment group is not significantly different from negative control (*p* > 0.05). Negative control denotes diluent buffer (sterile water), and Positive control denotes hydrogen peroxide (200 µM).

Understanding NP-induced oxidative stress in human cells is key to developing potent antibacterials that are also biocompatible for human use. Reactive oxygen species (ROS) may be generated upon NP exposure. Lipid peroxidation occurs when excess ROS affects cell membranes and can also lead to oxidation and denaturation of proteins and DNA damage, further inducing inflammatory immune responses and cell death^26^. Our results show no lipid peroxidation (MDA) in human lung epithelial (H-6053; Cell Biologics) and dermal fibroblast (106-05A-1526; Millipore Sigma) cells upon NH_2_–AgNPs treatments as the MDA levels were all below the background concentrations (negative control, dilution buffer). Rather, data revealed a decrease in MDA with up to 10 µg/mL of NH_2_–AgNPs treatments in lung cells compared to treatments with the negative control (dilution buffer) or the positive control (hydrogen peroxide) (Fig. 6). Such an ability to quench oxidative stress response makes NH_2_–AgNPs a biocompatible antibacterial candidate.

AgNPs have been reported to alter homeostasis and membrane permeability in *E. coli* and *Vibrio cholera*^42^. In a separate study, Kim et al.^43^ documented AgNPs to form pits within the cell membrane, thereby increasing cell permeability. Another study reported antibacterial potential of AgNPs synthesized using a mushroom (*Pleurotus sajor-caju*) extract, but the surface moiety was not determined in the study^44^. Research also indicates Ag^+^ ions could inhibit bacterial growth by disrupting DNA replication and inactivating thiol groups in many enzymes^43^. Bruins et al.^45^ suggested that metals could interact with cellular components through covalent and ionic bondings. At higher exposure levels, essential metals including Co, Cu, Ni, and Zn can also inhibit microbial growth and function^45,46^. Detail characterizations of surface functionalized AgNPs before and after interactions with the *E. coli* cells are paramount to better elucidate how these NPs could elicit toxicity to bacteria and other pathogenic microbes of human health importance. Employing electron microscopy (Figs. 4, 5, 6) coupled with surface spectroscopic techniques (Figs. 3, 4, 5, Supplementary Information Fig. S2) allowed us to confirm our in vitro* E. coli* toxicity results (Fig. 2).

Designing small 5 nm diameter NH_2_–AgNPs with high positive surface charge is found to be significantly inhibitory to *E. coli* compared to the larger size 45 nm Citrate–AgNPs with high negative surface charge. The bactericidal effects of NH_2_–AgNPs and the non-toxic effects of Citrate–AgNPs lend credence to the hypothesis that smaller size and highly positive surface charged AgNPs could serve as a next generation antibacterial agent to addressing the growing HAIs and patient health and safety.

## Summary

AR/MDR is widespread as new AR bacteria and mechanisms are being documented globally, undermining our ability to treat common bacterial diseases using current antibiotics^46^. Addressing HAIs due to increasing AR/MDR amongst bacteria is paramount for patient health and safety, and reducing hospital stay and associated health care cost^1–3^. Designing NPs with distinct particle properties allowed us to test the hypothesis that smaller size and highly positive surface charged AgNPs could serve as a next generation bactericidal agent. Our results show that small 5 nm diameter NH_2_–AgNPs with high positive surface charge was significantly inhibitory to *E. coli* compared to the larger size 45 nm Citrate–AgNPs with high negative surface charge. Further, results showed NH_2_–AgNPs inhibited adherent fimbriae expression and were bactericidal, while Ag^+^ ions were bacteriostatic but led to potentially resistant hexagonal honeycomb-like phenotype in *E. coli* dh5a. NH_2_–AgNPs were also found to quench oxidative stress response in human lung epithelium and skin fibroblasts, suggesting NH_2_–AgNPs’ potential application as a safer antibacterial candidate. Taken together, this study shows that ENM can be specifically tailored to render higher toxicity against the AR/MDR bacteria such as *E. coli* and to mitigate growing HAIs as current antibiotics are losing their efficacy^47^.

## Supplementary Information

Supplementary Information.

## Data Availability

All data that support the findings of this study are presented in the main text and the Supplementary Information.
