# Supplementary material for: Five nanometer size highly positive silver nanoparticles are bactericidal targeting cell wall and adherent fimbriae expression
Source: Sci Rep. 2022 Apr 25;12:6729. doi: 10.1038/s41598-022-10778-9 (PMC9039075; doi:10.1038/s41598-022-10778-9)
Supplement: Supplementary file 1 — Supplementary Information. [file 41598_2022_10778_MOESM1_ESM.docx]

**Supplementary Information**

**Title:** **Five nanometer size highly positive silver nanoparticles are bactericidal targeting cell wall and adherent fimbriae expression**

Lok R. Pokhrel^1,*^, Zachary L. Jacobs^3^, Dmitriy Dikin^4^, Shaw M. Akula^5^

^1^Department of Public Health, The Brody School of Medicine, East Carolina University, Greenville, NC, USA.

^2^School of Law, University of California, Berkeley, Berkeley, CA, USA.

^3^Department of Mechanical Engineering, College of Engineering, Temple University, Philadelphia, PA, USA.

^4^Department of Microbiology and Immunology, The Brody School of Medicine, East Carolina University, Greenville, NC, USA.

*Correspondence: LR Pokhrel, Phone: 252-737-5587; Email: [pokhrell18@ecu.edu](mailto:pokhrell18@ecu.edu).

**Table S1.** Representative protocol for the purification of AgNPs using the Tangential Flow Filtration (TFF) system.

| **Purification of unclean AgNPs** | **Electrical Conductivity (µS/cm)** |
| --- | --- |
| Started Volume = 500 ml | 1095 |
| Ended Volume = 70 ml | 1162 |
| Volume increased to 500 ml adding Milli-Q water | 185 |
| Ended Volume = 100 ml | 283 |
| Volume increased to 500 ml adding Milli-Q water | 36 |
| Ended Volume = 75 ml | 68 |
| Volume increased to 500 ml adding Milli-Q water | 11 |
| Ended Volume = 150 ml | 20 |
| Volume increased to 500 ml adding Milli-Q water | 10^*^ |

* obtained as purified AgNPs suspension with electrical conductivity of 10 µS/cm.

**Table S2.** Impact of incubation time (72 h) and temperature (35 °C) on the stability of nanoparticles in the carrier medium, i.e., moderately hard water (MHW), evaluated by measuring average hydrodynamic diameter (HDD) and zeta potential.

| **Parameters** | **Citrate-AgNPs** | | **NH_2_-AgNPs** | |
| --- | --- | --- | --- | --- |
|  | **Pre-incubation** | **Post-incubation** | **Pre-incubation** | **Post-incubation** |
| HDD ± SD (nm)^*^ | 11.0 ± 0.7 | 11.2 ± 1.3 | 4.3 ± 0.8 | 5.1 ± 0.3 |
| Zeta potential (mV) | -23.47 | -18.41 | +41.6 | +39.2 |

* Volume weighted hydrodynamic diameter (HDD) measured using DLS, and all size measurements were 100% by volume.

**Table S3** General linear model showing significant association between *E. coli* cell length and diameter (top table), followed by model parameters (bottom table).

| **Dependent Variable: Cell Length** | | | | |  | | |  |  | | |
| --- | --- | --- | --- | --- | --- | --- | --- | --- | --- | --- | --- |
| **Source** | **Type III Sum of Squares** | | **df** | | **Mean Square** | | | **F** | **Sig.** | | |
| Model | 2006.904^a^ | | 1 | | 2006.904 | | | 1386.473 | .000 | | |
| Diameter | 2006.904 | | 1 | | 2006.904 | | | 1386.473 | .000 | | |
| Error | 396.612 | | 274 | | 1.447 | | |  |  | | |
| Total | 2403.516 | | 275 | |  | | |  |  | | |
| a. R Squared = .835 (Adjusted R Squared = .834) | | | | | | | | |  | | |
| b. Computed using alpha = .05 | | | | | | | | | | | |
| Parameter | B | Std. Error | | t | | Sig. | 95% Confidence Interval | | | | |
|  |  |  |  |  |  |  | Lower Bound | | | Upper Bound | |
| Diameter | 2.924 | .079 | | 37.235 | | .000 | 2.769 | | | 3.079 | |
| a. Computed using alpha = .05 | | | | | | | | | | |  |


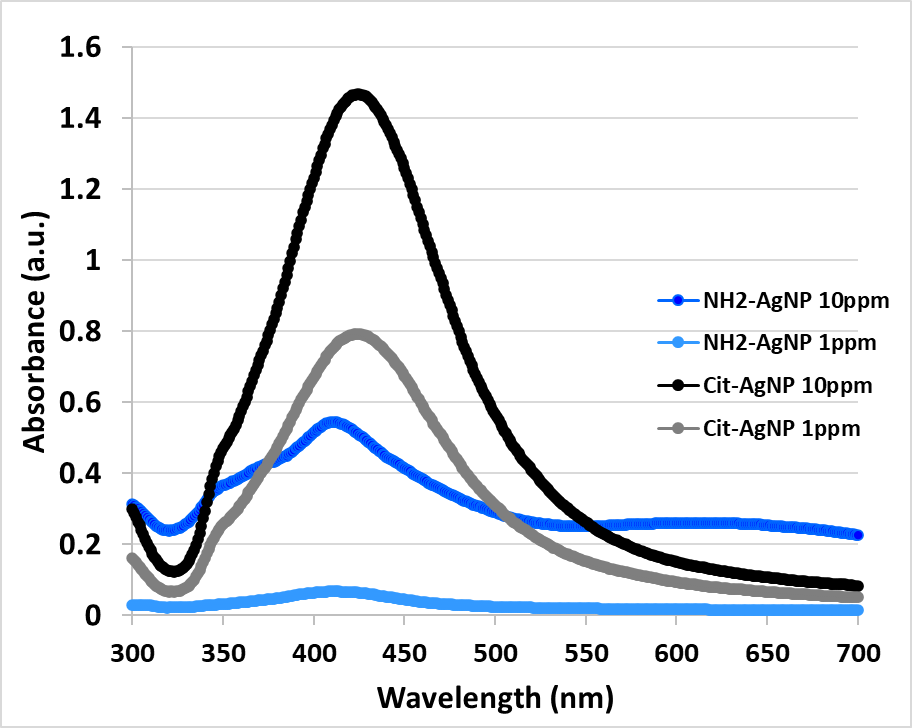


**Fig. S1.** UV-Visible spectra of purified NH_2_-AgNPs (1 µg/mL and 10 µg/mL) and Citrate-AgNPs (1 µg/mL and 10 µg/mL) used in this study showing no change in localized surface plasmon resonance (λ_max_) of both the AgNPs with dilution in Milli-Q water.

**
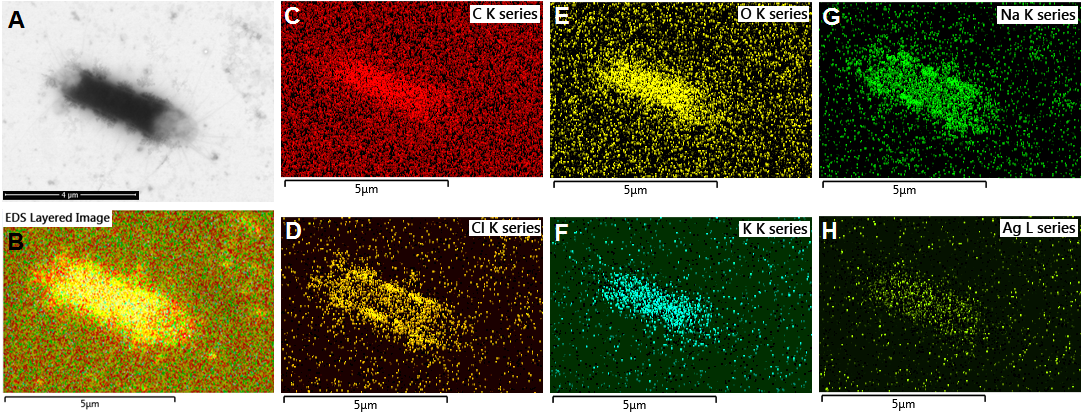
**

**Fig. S2.** Energy dispersive spectroscopy (EDS) elemental mapping of silver and other background elements (C, O, Na, Cl, and K) on the *E. coli* surfaces upon exposure to 10 µg/mL Citrate-AgNPs for 72 h. Scale bar denotes 5 µm for all images, except for A (scale bar = 4 µm).
